# Frequency of Electronic Personal Health Record Use in US Older Adults: Cross-Sectional Study of a National Survey

**DOI:** 10.2196/71460

**Published:** 2025-07-28

**Authors:** Lavlin Agrawal, Richelle Oakley DaSouza, Pavankumar Mulgund, Pankaj Chaudhary

**Affiliations:** 1 Department of Business Information Systems and Analytics, Willie A. Deese College of Business and Economics, North Carolina Agricultural and Technical State University, 1601 E. Market Street, Greensboro, NC, 27411, United States, 1 336-285-3394; 2 Department of Management Information Systems, Fogelman College of Business & Economics, University of Memphis, Memphis, United States

**Keywords:** electronic personal health record, aging population, self-efficacy, technology adoption, health IT, patient engagement

## Abstract

**Background:**

Electronic personal health records (ePHRs) hold significant potential to improve health management for older adults by enhancing access to medical information and facilitating communication with health care providers. However, usage remains low among individuals aged 65 and older. While existing research has identified barriers such as low self-efficacy, limited digital literacy, and usability challenges, the specific factors influencing the use of ePHRs among older adults are not yet fully understood.

**Objective:**

This study integrates the Aging and Technology framework with the Patient Technology Acceptance Model to examine key predictors of ePHR use among older adults, including age, education, issue involvement, performance expectancy, effort expectancy, and self-efficacy, while controlling for demographic factors such as gender, race, and income.

**Methods:**

This study utilizes data from the Health Information National Trends Survey (HINTS 5 cycle 3), which includes 532 respondents representing 13,136,180 US adults aged 65 years and older, after applying survey weights. Structural equation modeling was used to analyze the factors influencing the frequency of ePHR access over the past 12 months.

**Results:**

The findings indicate that older adults with higher self-efficacy used ePHRs more frequently. Additionally, issue involvement, performance expectancy, and effort expectancy were positively associated with ePHR use. Notably, self-efficacy partially mediated the relationship between age and the frequency of ePHR use.

**Conclusions:**

These findings suggest that enhancing self-efficacy, improving usability, and increasing the perceived benefits of ePHRs are critical for boosting usage among older adults. The study underscores the need for targeted interventions to support older users, simplify digital interfaces, and provide accessible educational resources, ultimately contributing to better health outcomes and an improved quality of life for older adults.

## Introduction

### Background

Health information technologies (HITs), such as electronic health records (EHRs) and electronic medical records (EMRs), are pivotal tools in modernizing health care by enhancing efficiency and improving the quality of care [1-3]. Beyond these general applications, HIT has the potential to address the unique medical needs of the older population. Extensive research demonstrates that HIT use can significantly improve health outcomes for older adults [4], particularly through tools such as EMRs and electronic personal health records (ePHRs). For example, EMRs in hospital settings help health care professionals identify at-risk patients, such as those prone to falls, by analyzing factors such as age and medication use [5]. Similarly, ePHRs empower older adults by providing easier access to health information, fostering improved communication with health care providers, and encouraging active engagement in personal health care management [6].

Luo et al [7] highlighted that, among older adults, the use of ePHRs was positively associated with the ease of understanding health information. ePHRs enable patients to track their health history to better understand the progress or deterioration of their health conditions. Patients with access to their health information experience increased medical data transparency, which reduces medical errors, improves trust in care providers, and enhances patient satisfaction [8]. As such, in the United States, the adoption of ePHRs has steadily increased. This growth has been supported by policies such as the Health Information Technology for Economic and Clinical Health Act of 2009 [9], which incentivized health care providers to adopt EHR systems through financial rewards, indirectly creating an infrastructure supportive of ePHRs. The subsequent Meaningful Use Program set specific objectives for health care providers to demonstrate effective use of EHRs, including patient-access capabilities. Further support came from the 21st Century Cures Act of 2016 [10], which enhanced patient access to electronic health information and prohibited information blocking, thereby facilitating smoother integration and utilization of ePHRs.

While the benefits of HIT for older persons are well-documented, the adoption and sustained use of these technologies face notable barriers, such as lower levels of technology usage among older adults [11]. Recent data indicate that approximately 4 in 10 individuals aged 65 years and older utilize ePHRs, a rate significantly lower than that of younger populations [12]. Furthermore, a previous study reported that approximately 54% of US adults have been offered access to their online medical records, and among those offered, 57% accessed them at least once, translating to about 31% of the adult population accessing their online medical records [13]. However, specific statistics for individuals aged 65 years and older are not provided in this source. Given that older adults are generally less likely to use the internet, it is reasonable to infer that their rates of ePHR usage may be lower than those of younger populations [12].

Further, researchers have found that older age is negatively associated with HIT [14]. Reduced cognitive and motor abilities, common among older adults, often complicate interactions with modern digital applications [15,16]. van der Vaart et al [8] discussed how older age is associated with the nonuse of web-based portals. Czaja and Lee [17] argued that although older adults are willing to use technology, many report usability problems with existing systems, which may, in part, be due to the cognitive and perceptual demands placed on the user. Their findings show that self-efficacy is an important predictor of general use of technology and that people with lower self-efficacy are less likely to use technology. However, individual-level barriers such as confidence and usability are only part of the picture. A growing body of research suggests that technologies are often not designed with the specific needs of older adults in mind [18,19]. Studies have also shown that older patients may not be offered digital health tools for various reasons, including—but not limited to—age-related stereotypes held by health care professionals or concerns about overburdening older patients [20,21]. Together, these individual and environmental barriers create a complex landscape that can inhibit older adults’ use of health technologies.

Despite advancements in technology, significant gaps remain in understanding the factors that influence the regular use of ePHRs among older adults. Existing research has focused on general barriers and facilitators, but limited knowledge exists about the interplay between these factors and how they shape the frequency of ePHR usage. This knowledge gap calls for a deeper examination of the factors affecting ePHR use in older populations. Additionally, limited electronic health literacy remains a critical challenge that hinders ePHR use in older populations [22]. However, the potential benefits cannot be overlooked. ePHRs offer older adults opportunities to coordinate their health care by sharing critical health information with providers and other stakeholders, an approach particularly crucial for managing chronic conditions that require multifaceted treatment strategies [7].

There are still unanswered questions about how older adults use ePHRs, particularly regarding the specific factors that facilitate or hinder ePHR use and the extent to which these factors influence it. To address this gap, this study explores the following research questions:

- What are the key factors influencing the use of ePHRs among older adults?
- How do age and education mediate the impact of self-efficacy on the frequency of ePHR use in older populations?
- What roles do facilitators such as issue involvement, performance expectancy, and effort expectancy of ePHRs play in encouraging regular use?

To answer these questions, we build on established theoretical frameworks such as the Patient Technology Acceptance Model (PTAM) [23], which provides insights into technology adoption and use processes. We extend the PTAM by incorporating factors specifically relevant to older persons, such as usability challenges, limited education, and issue involvement, to develop a comprehensive understanding of the dynamics at play. This study aims to contribute to the literature by offering empirical evidence and actionable insights into how ePHRs can be effectively designed and implemented to support the unique health care needs of aging populations.

### Hypotheses and Proposed Model

#### Overview

This study aims to deepen the understanding of the frequency of PHR use among the older population by addressing critical individual factors that influence usage. A nuanced understanding of these factors is essential for improving ePHR-related outcomes, such as patient empowerment, health care accessibility, and overall health management. Specifically, this research investigates the determinants influencing the frequency of ePHR use among individuals aged 65 years and older.

Based on the existing literature, we contend that theoretical frameworks grounded in the Technology Acceptance Model (TAM) [24], including the PTAM [23], are particularly suitable for investigating frequency of use, aligning well with our study’s specific outcomes. Previous research applying the TAM has effectively examined ongoing usage rather than merely initial adoption. For instance, McCloskey [25] utilized the TAM to evaluate sustained electronic commerce (e-commerce) behaviors, specifically the frequency of online purchasing, and found that perceived usefulness directly influenced the frequency of technology use. Similarly, Lederer et al [26] applied the TAM to explore frequent usage of the World Wide Web, explicitly addressing ongoing use rather than initial acceptance. Moreover, Martín-García et al [27] extended the TAM framework to examine older adults’ frequency of using technological devices, demonstrating the applicability of TAM constructs beyond initial intentions to sustained technological engagement.

Building upon this foundation, we integrate Czaja et al’s [28] conceptual model of aging and technology with the PTAM [23]. Our selection of the PTAM specifically addresses our research context—older patients managing multiple chronic conditions at home using digital health tools. Unlike broader acceptance models such as the TAM [24], the Unified Theory of Acceptance and Use of Technology (UTAUT) [29], and the Health Belief Model [30], PTAM explicitly considers patient-centric variables critical for older populations managing complex health conditions.

For example, while the TAM broadly emphasizes general technological acceptance through perceived ease of use and perceived usefulness, it lacks explicit patient-oriented or health outcome considerations [31]. Similarly, the UTAUT primarily addresses technology acceptance within general workplace contexts rather than the nuanced health care needs of older patients with chronic conditions [32]. The Health Belief Model likewise falls short by inadequately addressing the interactive dynamics unique to older patient populations [33].

By contrast, PTAM’s incorporation of patient-specific constructs—such as perceived health improvements, patient-provider relationships, and health care contextual factors—offers a more precise and comprehensive understanding of older patients’ sustained engagement with digital health interventions. This tailored approach provides richer theoretical insights and clearer practical implications, significantly enhancing explanatory power and applicability compared with general acceptance models.

#### Age

The utilization of ePHRs and patient portals exhibits notable age-related disparities. Despite being frequent consumers of health care services, older adults are significantly less likely to use these digital tools compared with younger individuals [34]. This gap in use can be attributed to age-related barriers, such as limited digital literacy and difficulties in navigating technological interfaces, which become increasingly pronounced with age [34,35].

A systematic review of patient acceptance of HIT underscored this trend, finding a negative correlation between age and HIT acceptance [14]. Similarly, Heart and Kalderon [36] provided compelling evidence that older adults demonstrate lower use of health-related information and communication technologies. Additionally, van der Vaart et al [8] highlighted that older individuals are generally less likely to use patient web portals. Based on these findings, we propose the following hypothesis:

- *H1a: Age is negatively associated with the frequency of ePHR use.*

Research has consistently shown that as individuals age, their electronic self-efficacy—the belief in their ability to effectively use technology—tends to decline. This decrease in self-efficacy is often attributed to a combination of factors, including reduced exposure to new technologies, cognitive decline, and negative stereotypes associated with older adults and technology use. For example, studies suggest that older adults are more likely to perceive technology as complex and overwhelming, which can lower their confidence in using digital tools such as EMRs or patient portals [28]. With age, physiological capacity and the ability to respond to environmental stresses decline, further contributing to the reduction in electronic self-efficacy [37]. Hence, we hypothesize:

- *H1b: Age is negatively associated with self-efficacy.*

#### Education

Smith et al [38] argued that individuals with higher levels of education are more likely to register for and utilize patient portals. Empirical evidence supports this claim, indicating that higher educational attainment is positively correlated with increased patient portal usage [39]. Educated individuals are more likely to engage with digital platforms for managing their health information, as they typically possess greater health literacy and familiarity with technology. Furthermore, a recent literature review highlights that lower educational levels act as a barrier to ePHR use [40]. Similarly, individuals with higher education are more likely to perceive the benefits of ePHRs, leading to higher usage rates [41]. Based on these findings, we propose the following hypothesis:

- *H2a: Education is positively associated with the frequency of ePHR use*.

Research consistently demonstrates that education and training play a pivotal role in enhancing individuals’ electronic self-efficacy, particularly in the context of using digital tools such as patient portals and EMRs. Czaja et al [28] highlighted a positive association between education and self-efficacy, emphasizing the importance of foundational skills acquired through educational experiences. These skills, including problem-solving, critical thinking, and basic computer literacy, are critical for successfully navigating digital platforms [42]. Furthermore, education has been shown to empower individuals across all age groups to overcome barriers to technology use. Based on this, we propose the following hypothesis:

- *H2b: Education is positively associated with self-efficacy.*

#### Issue Involvement

Issue involvement in the health care domain refers to “how relevant a specific health issue is to a patient” [43,44]. A more involved patient typically has a severe health condition and frequently visits health care providers [45]. Ross et al [46] argued that issue involvement has a significant positive impact on the use of patient-accessible medical records. This finding is further confirmed by Angst and Agarwal [45] and Abdelhamid et al [43]. A more involved patient is more likely to use ePHRs, as they help patients better prepare for upcoming visits with physicians by enhancing their knowledge of their medical condition, increasing their sense of control, and allowing them to seek clarification about treatment. Hence, we hypothesize:

- *H3: Issue involvement is positively associated with the frequency of ePHR use.*

#### Performance Expectancy

Performance expectancy is defined as the degree to which a person feels that using a system will help them perform a job more efficiently [29]. In keeping with this understanding, we refer to performance expectancy as the degree to which the patient believes that using ePHRs helps them monitor their health. Venkatesh et al [29] theorized that performance expectancy drives the intention to use information systems. Several researchers have also identified performance expectancy as one of the critical predictors of eHealth acceptance and use [40,47-49]. Patient portals help improve patient engagement and empower individuals to access their health information anytime and anywhere [50]. Thus, we hypothesize:

- *H4: Performance expectancy is positively associated with the frequency of ePHR use.*

#### Effort Expectancy

Venkatesh et al [29] defined effort expectancy as the degree of comfort associated with system use. Consistent with Venkatesh et al [29], we define effort expectancy as the degree of ease associated with understanding the health information in the online medical record. Effort expectancy is another key variable that drives use intentions [46]. Many researchers have also identified effort expectancy as one of the critical predictors of health technology use [2,23,37,48]. Therefore, we also propose the following hypothesis:

- *H5: Effort expectancy is positively associated with the frequency of ePHR use.*

#### Self-Efficacy

Research has consistently demonstrated that electronic self-efficacy—the belief in one’s capability to effectively use technology—declines with age due to factors such as reduced exposure to new technologies, cognitive decline, diminished physiological capacity, and negative stereotypes about older adults’ technology use [28,37]. Consequently, older adults often perceive technology, including ePHRs, as complex and overwhelming, undermining their confidence in engaging with such digital tools. Therefore, we selected self-efficacy as a mediator to explicitly capture the psychological mechanism linking age with ePHR use. Rather than age directly limiting ePHR engagement, we posit that reduced electronic self-efficacy partially explains why older adults use ePHRs less frequently. This approach emphasizes the central role of self-efficacy in technology use among aging populations [36,51]. Hence, we hypothesize:

- *H6: Self-efficacy is positively associated with the frequency of ePHR use.*

**Figure 1.**
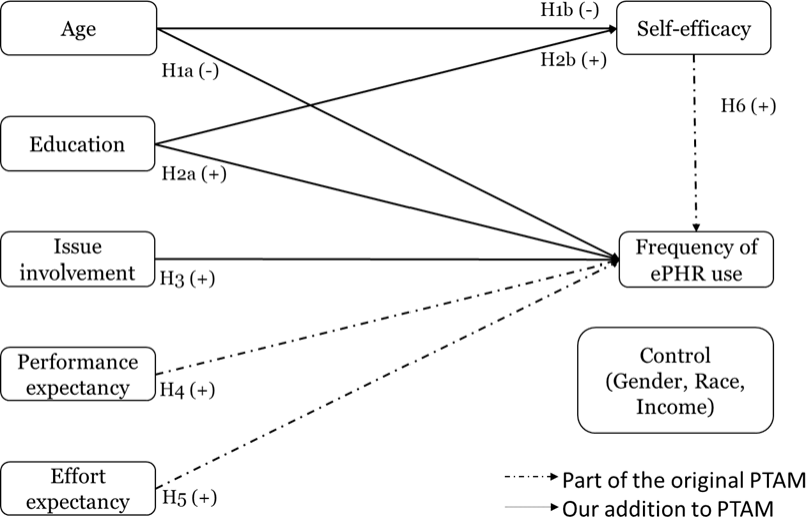
The research model. ePHR: electronic personal health record; PTAM: Patient Technology Acceptance Model.

Our proposed model is depicted in Figure 1. Additionally, we control for demographic factors such as gender, race, and income to account for their potential influence on the frequency of ePHR use.

## Methods

### Data Source

This study used publicly available cross-sectional data collected in 2019 by the National Cancer Institute (Health Information National Trends Survey [HINTS] 5 cycle 3). The cross-sectional analysis for this study was limited to a subset of the original dataset and included participants aged 65 years or older. The total number of individuals aged 65 years or older was 1961. Of these, 1234 had not used an ePHR at least once in the past 12 months and were therefore excluded from the analysis. Further, 195 responses were removed due to missing values for key variables. The final sample consisted of 532 respondents, representing 13,136,180 US adults aged 65 and older when survey weights were applied.

### Ethical Considerations

The HINTS 5 survey, conducted with the general population, underwent expedited review and received approval from the Westat Institutional Review Board on March 28, 2016 (project number 6048.14). This analysis used deidentified, publicly available data from HINTS, which did not constitute human research as defined by 45 CFR 46.102 and, therefore, did not require Institutional Review Board review.

### Measurements

In this study, the primary dependent variable, “frequency of ePHR use,” is operationalized using a single-item measure assessing how frequently patients accessed their online medical records within the past 12 months. The response options are categorized as follows: 1-2 times, 3-5 times, 6-9 times, and 10 or more times. Individuals who reported not accessing their online medical records during the past year were excluded from the analysis, as critical variables pertinent to these respondents were not captured.

Performance expectancy, representing the perceived usefulness of online medical records for health monitoring, is measured using a 5-point Likert scale, ranging from “5=very useful” to “1=don’t use.” Effort expectancy, reflecting the perceived effort necessary to understand health information in online medical records, is assessed using a 4-point Likert scale ranging from “4=very easy” to “1=very difficult.” Issue involvement captures an individual’s engagement with personal health management and is operationalized as the number of interactions a respondent has had with health care providers over the past year. Self-efficacy in accessing electronic health records is assessed through a binary-choice question indicating whether respondents have utilized any electronic method to access their medical records during the past 12 months.

Single-item measures are used for several constructs in this study. Such measures are considered acceptable when questions are clear, unambiguous, and not prone to multiple interpretations [52]. Additionally, single-item measures are prevalent and widely validated within information systems research, particularly in studies applying structural equation modeling (SEM) in health care contexts [43,44].

Gender, race, and income are incorporated as control variables, consistent with prior studies [43,44]. For comprehensive details on questionnaire items, scales, and specific variable operationalizations, please refer to Multimedia Appendix 1.

### Statistical Analysis

This study uses SEM to conduct a path analysis. Although SEM is predominantly used to model latent variables, it is also commonly applied to conduct a path analysis in mediation models. We examine 2 mediating relationships: first, self-efficacy mediates the relationship between age and the frequency of ePHR use; second, self-efficacy also mediates the relationship between education and the frequency of ePHR use. Accordingly, we use SEM to test the model, consistent with prior research [53,54]. We used SEM with weighted least squares mean and variance adjusted estimation to test the hypotheses. Weighted least squares mean and variance adjusted is well-suited for models with ordinal outcome variables [55,56]. The analysis was conducted in R (R Foundation) using the “lavaan.survey” package. We incorporated HINTS-supplied survey weights and used jackknife variance estimation techniques to account for the complex HINTS sampling design and to calculate nationally representative estimates [57].

## Results

### Descriptive Statistics

Table 1 shows the descriptive statistics of the survey respondents. The survey included questions about the frequency of participants’ ePHR use, as well as questions related to the model variables, including performance expectancy, effort expectancy, issue involvement, self-efficacy, age, education, gender, race, and income.

**Table 1.** Descriptive statistics.

Demographic characteristics		Sample size (N=532)^a^	With survey weights (N=13,136,180)^b^
Gender			
Male		249 (46.8)	5,729,371
Female		283 (53.2)	7,406,809
Race			
White		458 (86.1)	11,961,412
Non-White		74 (13.9)	1,174,768
Education			
Less than high school		7 (1.3)	278,445
High school or higher		525 (98.7)	12,857,735
Income (US $)			
Less than 20,000		57 (10.7)	1,191,896
20,000 to <35,000		68 (12.8)	2,009,787
35,000 to <50,000		86 (16.2)	2,100,148
50,000 to <75,000		122 (22.9)	3,110,545
75,000 or higher		199 (37.4)	4,723,804
Age (years)			
Range		65-97	65-97
Mean (SD)		71.68 (5.66)	71.90 (6.09)

a The sample size for gender, race, education, and income is presented as n (%).

b Data are presented as n for gender, race, education, and income.

### Reliability and Validity

Table 2 presents the correlations between all variables of interest. Correlation coefficients are important because high correlations among independent variables may indicate multicollinearity, which can introduce bias into the model results. Multicollinearity is not a concern in this analysis, as all correlations fall within the acceptable threshold of 0.6 [58], with the highest correlation being 0.37 between performance expectancy and effort expectancy. Table 2 also provides the means and SDs for the principal variables.

**Table 2.** Correlation matrix.

Correlation	Mean (SD)	Performance expectancy	Effort expectancy	Issue involvement	Self-efficacy
Performance expectancy	4.15 (1.03867)	1.00	—^a^	—	—
Effort expectancy	3.30 (0.67763)	0.37	1.00	—	—
Issue involvement	3.80 (1.49733)	−0.01	−0.14	1.00	—
Self-efficacy	0.82 (0.38126)	0.17	0.06	0.09	1.00

a Not applicable.

### Common Method Variance

As data are self-reported and collected through a single survey, they may suffer from common method variance (CMV), which hampers the relationship between the variables [59]. We used the marker variable technique [60] to check if the data are suffering from CMV. Marker variable is a variable that is theoretically unrelated to 1 or more of the principal variables measured in the study and typically has a low correlation with the central variables.

The theoretically unrelated construct “morning person or night person” was used as a marker variable. The correlations between the marker variable morning person or night person and other principal variables were below the threshold of 0.1 (performance expectancy: −0.08; effort expectancy: −0.08; self-efficacy: 0.03) [60], except for issue involvement (0.17). The low correlation of the marker variable with the principal variables in the model indicates that CMV is not a problem.

### Data Analysis

As the National Cancer Institute administered both a paper-based questionnaire and an online questionnaire to survey participants, we first assessed whether the mode of survey introduced any biases. We regressed the dependent variable, “frequency of ePHR use,” on the mode of survey and found that the relationship between the 2 was nonsignificant (*P*=.48). Thus, the mode of survey administration did not introduce any bias in the main outcome variable, that is, “frequency of ePHR use.”

The SEM results are presented in Table 3. The overall fit statistics (*χ*^2^_17_=13.230, *P*=.004; Comparative Fit Index=0.950, Tucker-Lewis Index=0.715, root-mean-square error of approximation=0.080, standardized root-mean-square residual=0.022) indicate a good model fit [61]. Table 4 presents the results of the mediation analysis for self-efficacy with age and education.

The results show that age is negatively associated with self-efficacy (*β*=−.9890, *P*=.03), suggesting that as age increases, self-efficacy decreases, supporting H1b. However, no significant (*P*=.85) relationship was found between self-efficacy and education (H2b) in our research context.

Further, the results show that age is positively associated with the frequency of ePHR use (*β*=1.4950, *P*=.03), suggesting that as age increases, ePHR use also increases. This is the opposite of what we hypothesized for H1a. This counterintuitive trend may reflect increased health care needs among older adults, leading to greater engagement with ePHRs for managing chronic conditions, medication schedules, and communication with physicians. No significant (*P*=.88) relationship was found between education and the frequency of ePHR use (H2a) in our research context. H3 proposed a positive relationship between issue involvement and the frequency of ePHR use. The path coefficient was positive and statistically significant (*β*=.2560, *P*<.001), suggesting that higher issue involvement leads to higher ePHR use.

Our analysis also revealed a significant positive relationship between performance expectancy and the frequency of ePHR use (*β*=.2470, *P*<.001), as well as between effort expectancy and the frequency of ePHR use (*β*=.1850, *P*=.04). These findings suggest that higher performance expectancy and higher effort expectancy both lead to increased ePHR use, supporting H4 and H5. We also found that self-efficacy is positively associated with the frequency of ePHR use (*β*=.4990, *P*<.001), supporting H6.

Further analysis confirms that self-efficacy partially mediates the relationship between age and the frequency of ePHR use among older adults, as evidenced by the statistical significance of both indirect (*P*=.03) and direct effects (*P*=.03). Specifically, self-efficacy accounts for 49.2% (−0.493/1.002) of the total effect, underscoring its substantial role as a mediator in this relationship. However, we did not find any statistically significant mediating effect of self-efficacy on the relationship between education and the frequency of ePHR use.

**Table 3.** Results of structural equation modeling.

Dependent variable and hypothesis	Variables	Estimate	95% CI	*P* value	Significant
Self-efficacy					
H1b	Log (age)	−0.9890	−1.8870 to −0.0900	.03	Yes
H2b	High school or more	−0.0360	−0.4070 to 0.3350	.85	No
Control	Female	−0.0190	−0.1300 to 0.0910	.73	No
Control	White	−0.0040	−0.1460 to 0.1370	.95	No
Control	Income	0.0120	−0.0200 to 0.0430	.47	No
Dependent variable: frequency of electronic patient health record use					
H1a	Log (age)	1.4950	0.1460 to 2.8440	.03	Yes
H2a	High school or more	0.0320	−0.3770 to 0.4410	.88	No
H3	Issue involvement	0.2560	0.1810 to 0.3300	<.001	Yes
H4	Performance expectancy	0.2470	0.1340 to 0.3600	<.001	Yes
H5	Effort expectancy	0.1850	0.0070 to 0.3640	.04	Yes
H6	Self-efficacy	0.4990	0.3260 to 0.6730	<.001	Yes
Control	Female	0.1970	−0.0410 to 0.4340	.10	No
Control	White	0.0590	−0.3050 to 0.4220	.75	No
Control	Income	−0.0300	−0.1290 to 0.0680	.54	No

**Table 4.** Mediation results of structural equation modeling.

Variables	Estimate	95% CI	*P* value	Significant
Log (age)				
Indirect through self-efficacy	−0.4930	−0.9410 to −0.0460	.03	Yes
Direct	1.4950	0.1460 to 2.8440	.03	Yes
Total	1.0020	−0.3170 to 2.3200	.14	No
High school or more				
Indirect through self-efficacy	−0.0180	−0.2040 to 0.1680	.85	No
Direct	0.0320	−0.3770 to 0.4410	.88	No
Total	0.0140	−0.4860 to 0.5140	.96	No

## Discussion

### Determinants of ePHR Use

Our study aimed to update the PTAM by examining the impact of performance expectancy, effort expectancy, and self-efficacy on the frequency of ePHR Use. By using an integrated framework, this research provides actionable insights into the factors driving ePHR usage among older adults, incorporating variables that have not been widely explored in prior studies. This study makes both a significant theoretical and practical contribution.

### Theoretical Implications

This study contributes to the literature on ePHR use by building on and extending existing theoretical frameworks, including the Aging and Technology framework [28] and the PTAM [23]. Prior research has predominantly highlighted barriers to ePHR use among older adults, such as reduced self-efficacy, usability challenges, and low digital literacy [15,17]. While these studies provide an important foundation, our findings challenge and refine these perspectives by demonstrating a positive association between age and ePHR use, with self-efficacy serving as a partial mediator in this relationship.

Contrary to earlier work suggesting older age is a barrier to HIT use [8,14], this study reveals that age positively influences ePHR use when health needs increase, as chronic illnesses and frequent health care interactions necessitate greater reliance on digital tools. This finding aligns with studies emphasizing the contextual nature of technology use, where health-related motivations can offset age-related challenges [7]. By demonstrating that older adults use ePHRs more frequently despite lower self-efficacy, this study extends theoretical models by incorporating health-related drivers, such as issue involvement, into the broader narrative of technology adoption.

Self-efficacy, identified as a strong predictor and mediator, validates and expands prior work in the field. Studies have consistently highlighted self-efficacy as a key determinant of technology use [62]. Our findings reinforce this and reveal that self-efficacy mediates the impact of both age and education on ePHR use. This underscores the critical role of psychological factors, particularly in populations facing cognitive and physical challenges, and aligns with theories from the PTAM [23] and the UTAUT [29].

The study also affirms the relevance of performance expectancy and effort expectancy as critical drivers of ePHR usage, consistent with the PTAM and UTAUT frameworks [47,48]. However, by identifying issue involvement as a significant predictor, this study contributes a novel dimension to the literature. This finding supports prior arguments that perceived relevance and personal health involvement significantly enhance engagement with health technologies [43,45].

Overall, this research advances theoretical understanding by integrating psychological, motivational, and contextual factors into established frameworks for HIT use. It bridges gaps in the existing literature by demonstrating that age, when considered alongside mediating factors such as self-efficacy and health involvement, can positively influence ePHR use. These insights offer a nuanced understanding of older adults’ interactions with health technologies, offering a robust foundation for future research and practical interventions.

### Practical Implications

#### Overview

The findings of this study underscore several actionable strategies for health care providers, policy makers, and technology developers to enhance ePHR usage among older adults. By addressing barriers and leveraging the facilitators identified, targeted interventions can empower older adults to better manage their health through ePHRs, contributing to improved health outcomes and broader system-wide benefits, as outlined below.

#### Building Self-Efficacy Through Tailored Training Programs

To enhance older adults’ self-efficacy in using ePHRs, health care organizations can establish targeted training and education programs aligned with the recommendations of the Office of the National Coordinator for Health Information Technology [63]. These programs could include workshops in collaboration with senior community centers and local health care providers, focusing on essential tasks such as medication tracking, viewing test results, and scheduling appointments [64]. Additionally, peer-led mentoring initiatives, consistent with community-based health literacy efforts promoted by Healthy People 2030, can encourage skill sharing among older adults [65].

#### Supporting Continuous Improvement and User-Centered Innovation

Continuous evaluation through user feedback mechanisms should be institutionalized to guide the ongoing improvement of ePHR technologies [66], particularly for older adults. Actively encouraging the participation of older adults in innovation processes can help health care organizations advance the user-centered innovation strategies promoted by federal health technology initiatives [63]. Collaboration among academia, health care providers, and technology developers can further drive innovation tailored to the evolving needs of older adults.

#### Promoting Health Involvement Through Guided Support

Health care providers can encourage older adults’ active participation in managing their health through targeted education campaigns that highlight the practical benefits of ePHRs. Public health strategies that emphasize patient-provider communication, administrative ease, and improved health care outcomes can be strengthened by incorporating user testimonials and success stories [67]. Additionally, guided tutorials embedded within ePHRs can further support patient engagement, aligning with strategies recommended by the Agency for Healthcare Research and Quality for patient empowerment [68].

Implementing these recommendations, grounded in existing US health care policies and strategies, can significantly enhance ePHR adoption among older adults, help reduce health disparities, and promote a more efficient and patient-centered health care system.

### Limitations

This study has several limitations that should be considered. First, the use of secondary data from the HINTS limited the analysis to variables available in the dataset. Important factors, such as detailed measures of digital literacy or prior technology experience, could not be examined. Further, this study included only individuals who reported using ePHRs at least once in the past 12 months, as key variables of interest were not captured for those who had not used ePHRs. Additionally, some variables in this study were measured using single-item scales, which may lack the robustness of multi-item measures. However, single-item measures are considered acceptable when the constructs are straightforward and unambiguous [52]. We acknowledge that single-item measures inherently do not allow for traditional assessments of internal consistency reliability, which presents a limitation in this study. This limitation necessitates cautious interpretation of the findings. Future research should aim to incorporate multi-item scales where feasible and conduct reliability analyses to ensure the stability and consistency of these measures.

Second, the reliance on self-reported data introduces the possibility of CMV, which could inflate relationships between variables [59]. To address this concern, the study applied the marker variable technique [53], which confirmed that CMV was not a significant issue. Third, the cross-sectional design of the study limits the ability to establish causal relationships among the variables. Longitudinal research is needed to explore how factors such as self-efficacy and performance expectancy influence the use of ePHRs over time. Fourth, although the use of survey weights enhances the representativeness of the sample for the United States, the relatively small sample size remains a limitation that future research with a more targeted approach could address. Additionally, we did not control for factors such as income, digital literacy, and prior technology experience, as these variables were not accessible in the dataset. The absence of these controls may limit the completeness of the study. Future research should incorporate these variables to provide a more comprehensive understanding of the frequency of HIT use among older adults. Finally, the findings are specific to the US context and may not fully generalize to other populations or health care systems. Despite these limitations, the study offers valuable insights and lays important groundwork for future research.

### Conclusions

This study emphasizes the important role of ePHRs in empowering older adults to manage their health and maintain independence. By utilizing survey weights, the findings can be generalized to the broader US population, making them particularly relevant as the aging demographic continues to grow. Unlike previous research, this study reveals a positive relationship between age and the level of ePHR use, challenging the common assumption that older adults are reluctant to adopt health technologies.

Key factors driving ePHR usage are performance expectancy, effort expectancy, self-efficacy, and issue involvement, all of which offer actionable pathways for increasing the frequency of use among older adults. Raising awareness of the practical benefits and ease of use of ePHRs can encourage more frequent engagement. Additionally, addressing usability concerns and emphasizing the relevance of ePHRs to individual health needs can further promote their adoption. Frequent use of ePHRs empowers older adults to manage chronic conditions, access vital health information, and make informed decisions, ultimately enhancing their quality of life.

As health care systems strive to meet the challenges posed by an aging population, integrating ePHRs into routine care and ensuring equitable access can provide both social and economic benefits. This study lays the groundwork for targeted interventions aimed at bridging the digital divide and fostering a more inclusive, health-empowered society for older adults.

## Supplementary material

10.2196/71460 Multimedia Appendix 1 Operationalization of constructs.

## Abbreviations

CMV: common method variance
e-commerce: electronic commerce
EMR: electronic medical record
ePHR: electronic personal health record
HINTS: Health Information National Trends Survey
HIT: health information technology
PTAM: Patient Technology Acceptance Model
SEM: structural equation model
TAM: Technology Acceptance Model
UTAUT: Unified Theory of Acceptance and Use of Technology
