# Supplementary material for: Frequency of Electronic Personal Health Record Use in US Older Adults: Cross-Sectional Study of a National Survey
Source: JMIR Aging. 2025 Jul 28;8:e71460. doi: 10.2196/71460 (PMC12303404; doi:10.2196/71460)
Supplement: Multimedia Appendix 1 [file aging-v8-e71460-s001.docx]

## Appendix 1: Operationalization of Constructs (source HINTS 5 Cycle 3)

| **Construct** | **Question** | **Scale*** | **Coding*** |
| --- | --- | --- | --- |
| Extent of ePHR Use (EU) | How many times did you access your online medical record in the last 12 months? | 1: 1 to 2 times  2: 3 to 5 times  3: 6 to 9 times  4: 10 or more times | 1: 1 to 2 times  2: 3 to 5 times  3: 6 to 9 times  4: 10 or more times |
| Performance Expectancy (PE) | In general, how useful is your online medical record for monitoring your health? | 5: don't use  4: Not at all useful  3: Not very useful  2: Somewhat useful  1: Very useful | 1: don't use  2: Not at all useful  3: Not very useful  4: Somewhat useful  5: Very useful |
| Effort Expectancy (EE) | How easy or difficult was it to understand the health information in your online medical record? | 4: Very difficult  3: Somewhat difficult  2: Somewhat easy  1: Very easy | 1: Very difficult  2: Somewhat difficult  3: Somewhat easy  4: Very easy |
| Self-efficacy (SE) | In the past 12 months have you used a computer, smart phone, or other electronic means to look up medical test results? | 0: No  1: Yes | 0: No  1: Yes |
| Issue Involvement (II) | In the past 12 months, not counting times you went to an emergency room, how many times did you go to a doctor, nurse, or other health professional to get care for yourself? | 0: None  1: 1 time  2: 2 times  3: 3 times  4: 4 times  5: 5-9 times  6: 10 or more times | 0: None  1: 1 time  2: 2 times  3: 3 times  4: 4 times  5: 5-9 times  6: 10 or more times |
| Female | Are you male or female? | Female or Male | 1: Female  0: Male |
| Age | What is your age? |  |  |
| Education | What is the highest grade or level of schooling you completed? |  | 0: Up to High school 1: More than High school |
| Race | White |  | 0: Non-White  1: White |
| Morning-night person (MNP) | Which do you consider yourself to be? | 1: I’m definitely a morning-person  2: I’m more of a morning-person than a night-person  3: I’m neither a morning-person nor a night-person  4: I’m more of a night-person than a morning-person  5: I’m definitely a night-person | 1: I’m definitely a morning-person  2: I’m more of a morning-person than a night-person  3: I’m neither a morning-person nor a night-person  4: I’m more of a night-person than a morning-person  5: I’m definitely a night-person |
| Scale: it represents how questions’ option were provided in the survey.  Coding: It represents how these options were used in this analysis. | | | |
